# Zhoushi Qiling decoction inhibits proliferation of human prostate cancer cells through IL6/STAT3 pathway

**DOI:** 10.7150/jca.84943

**Published:** 2023-07-16

**Authors:** Hongwen Cao, Yigeng Feng, Peng Sun, Lei Chen, Dan Wang, Renjie Gao

**Affiliations:** Surgical Department I (Urology Department), LONGHUA Hospital Shanghai University of Traditional Chinese Medicine, No. 725 Wanping Road South, Xuhui District, Shanghai 200032, China.

**Keywords:** prostate cancer, Zhoushi Qiling decoction, proliferation, IL-6/STAT3

## Abstract

**Background:** Prostate cancer is the most common malignant tumor in men, accounting for one of the top five cancer incidences worldwide. However, there is no effective pharmacological treatment for advanced prostate cancer (APC). Herein, we aim to investigate the mechanism of Zhoushi Qiling decoction (ZQD), a traditional Chinese medicine compound, in inhibiting prostate cancer cells proliferation and tumor growth.

**Methods:** IC50 was determined in PC3 and DU145 cells. Cell viability was determined using MTT assay after interleukin (IL) 6 stimulation. Cell proliferation ability was evaluated using colony formation assay. IL-6/signal transducer and activator of transcription 3 (STAT3) signaling pathway was analyzed using qRT-PCR and Western blot in PC3 and DU145 cells and xenograft tumor tissues.

**Results:** It was found that ZQD significantly inhibited Il-6-induced cell viability and proliferation in PC3 and DU145 cells. Moreover, ZQD significantly reduced mRNA levels of IL-6, IL-1β, STAT3, Bcl2, and CyclinD1, stimulated by IL-6. The protein levels of p-STAT3, Bcl2 and CyclinD1 were reduced by ZQD treatment at 40 mg/mL both in PC3 and DU145 cells. Additionally, in xenograft tumor tissues, tumor volume, weight and proliferation were significantly reduced by ZQD treatment. In addition, the mRNA and protein levels of IL-6 and pSTAT3 were significantly inhibited by ZQD treatment in vivo.

**Conclusion:** We demonstrate that ZQD can effectively reduce cell proliferation and tumor growth by inhibiting the activation of IL-6/STAT3 signaling pathway.

## Introduction

Prostate cancer is the most common malignant tumor of the male genitourinary system, accounting for the top five incidences of cancer worldwide and the second highest incidence of malignant tumors in men worldwide 1,2. In United States, the incidence of prostate cancer ranks first among all male malignancies, accounting for approximately 30% of all male malignancies 3. The morbidity and mortality rates of patients with prostate cancer are increasing each year. Unfortunately, most prostate cancers are metastatic or locally advanced by the time they are diagnosed 4-7. The main treatments for advanced prostate cancer (APC) include radical prostatectomy, radiation therapy, endocrine therapy and active surveillance. While these treatments have improved survival rates, they have also had a significant impact on patients' physical and mental status and social functioning, interfering with their daily lives 8,9. The effectiveness of these treatments varies, as does the risk of mortality, recurrence, and complications. The research and development of optimal treatment for prostate cancer has been a heated issue worldwide.

Interleukin 6 (IL-6) is a glycoprotein consisting of 212 amino acids encoded by the IL-6 gene located on human chromosome 7p21-14 10. The receptors for IL-6 consist of the IL-6-specific receptor subunit (β chain) and the signal transducer gp130. IL-6 utilizes the Janus kinase signal transducer and activator of transcription (JAK-STAT) as the primary mediator of signal transduction, and the key step in facilitating transcription is controlled by activation of signal transducer and activator of transcription 3 (STAT3), a key component of the JAK-STAT pathway 11,12. Serum IL-6 levels were found to be elevated during denervation therapy, and high IL-6 expression may be involved in the malignant progression from hormone-sensitive to hormone-refractory prostate cancer 13,14. Liu et al. reported that the IL-6/STAT3 axis was involved in the development of enzalutamide resistance in prostate cancer 15. Prostate cancer cells become resistant to enzalutamide by producing IL-6, which leads to the activation of STAT3. Upregulation of STAT3 leads to a decrease in the sensitivity of prostate cancer cells to enzalutamide. Overexpression of active STAT3 in prostate cancer cells induces resistance to enzalutamide treatment 15.

Androgen-deprivation therapy is commonly used to treat patients with advanced prostate cancer. Patients then have a very poor prognosis and need more effective treatment. In our previous work, we combined ADT with the ZQD developed by Professor Zhou Zhiheng of Longhua Hospital, Shanghai University of Traditional Chinese Medicine, and found that it was effective in reducing the pain of endocrine therapy and prolonging the survival of patients.^16^ To investigate the mechanism related to ZQD treatment for prostate cancer, we treated human prostate cancer cell line DU145 with ZQD and found that miR-143 was significantly increased in the experimental group by microarray and found that ZQD promoted the apoptosis of DU145 16. In this study, we aim to further investigate the related mechanism of ZQD in inhibiting the proliferation of prostate cancer cells.

## Methods

### ZQD Treatment

The formulation of ZQD was used as described in our previous work 16: 15 g of Astragalus mongholicus Bunge (root), 30 g of Rabdosia rubescens Hara (root), 15 g of Radix Rehmanniae (root), 15 g of radix codonopsis (root), 9 g of rhizoma curcumae longae (root), 15 g of Solanumseptemlobum Bunge (seed), 15 g of Herba Leonuri (Herba), and 9 g of Radix Glycyrrhizae Preparata (Herba). Followingly, ZQD was boiled in 500 ml of sterile water, then continued to decoct on low heat for 10-15 min. After filtering, the liquid extraction of ZQD was desiccated to 1.14 g crude drug/mL, and stored at -80ºC.

### Cell culture

PC3 and DU145 cells were purchased from the ATCC (Manassas, VA) and cultured in Ham's F12 and DMEM medium (Gibco, Carlsbad, CA). Medium was supplemented with 10% fetal bovine serum (FBS) (Invitrogen, Carlsbad, CA). Cells were cultured at 37ºC in a humidified atmosphere in a 5% CO_2_ incubator. The cells were cultured with 40 mg/mL of ZQD with the culture medium changed every 2 days.

### MTT assay

6000 cells per well were plated in 96-well plates and treated with ZQD at concentrations of (5, 15, 30, 45, 60, 75, 90 mg/mL) for 24, 48, 72, and 96 h. Cell viability was determined using MTT assay 17 (Bio-Rad, Hercules, CA).

### Colony formation assay

2 ×10^3^ cells per well cells were seeded in a 6 cm culture dish. Cell proliferation was assessed using colony formation assay at 10 days after treatment. Colonies were washed twice with phosphate-buffered saline and fixed with methanol for 15 min. Next, cells were crystallized violet staining for 30 min 18.

### Xenograft tumor model

Male 8-week-old BALB/C nude mice were purchased from GemPharmatech (Nanjing, China). All animal experiments protocol were approved by LONGHUA Hospital Shanghai University of Traditional Chinese Medicine. After successful continuous ether inhalation anesthesia, a longitudinal incision was made in the lower abdomen. Bilateral seminal vesicles were located on the dorsal side of the bladder root, and prostate tissue were located at the root of the seminal vesicles. The cell line PC3 was mixed with matrix gel (Matrigel) at a ratio of 1:1. 20 μL of the tumor cells were injected in the prostate gland of each nude mouse with a cell count of 5×10^6^. The tumor cells were injected by gently piercing the prostate envelope with the tip of an insulin syringe. Subsequently, the incision was closed with absorbable sutures. When the tumor volume reached 50-100 mm^3^, mice were randomly assigned to three groups: control group (NC), low-dose ZQD group and high-dose ZQD group. Low-dose ZQD group was treated with 0.2 ml of a solution containing 200 mg of ZQD twice daily (10 g/kg). High-dose ZQD group were treated with 0.2 ml of a solution containing 400 mg of ZQD twice daily (20 g/kg).

### RT-qPCR

Total mRNA was isolated using Trizol (Invitrogen). cDNA was then synthesized using the High-Capacity cDNA Reverse Transcription Kit (Thermo Fisher, Waltham, MA) according to the its protocol. The mRNA expression relative change was determined using 2^-ΔΔCt^ method. The target mRNA was normalized to *GAPDH*.

### Western blot

Total protein was extracted using RIPA buffer (Beyotime, Shanghai, China). The concentrations of total proteins were determined using BSA assay kit (Beyotime). Followingly, proteins were separated on 10-20% SDS gel and transferred to a PVDF membrane. The blots were incubated with primary antibody against IL-6 (#66146-1-Ig, 1:1000, ProteinTech, USA), STAT3 (#10253-2-AP, 1:1000, ProteinTech, USA), p-STAT3 (#9131L, 1:500, Cell Signaling, USA), Bcl2 (#12789-1-AP, 1:1000, ProteinTech, USA), CyclinD1 (#ab16663, 1:1000, Abcam), β-actin (#ab8226, 1:1000, Abcam). The secondary IgG-HRP antibodies were used and protein bands were determined using ECL reagent (Beyotime). The bands were quantified using Image J software (NIH, USA).

### Statistical analysis

Data were presented as mean ± standard deviation (SD). Significance was determined using one- or two-way ANOVA followed a post hoc test. P < 0.05 was considered statistically significant.

## Results

### ZQD inhibited cell proliferation of PC3 and DU145 cells

**Figure 1:**
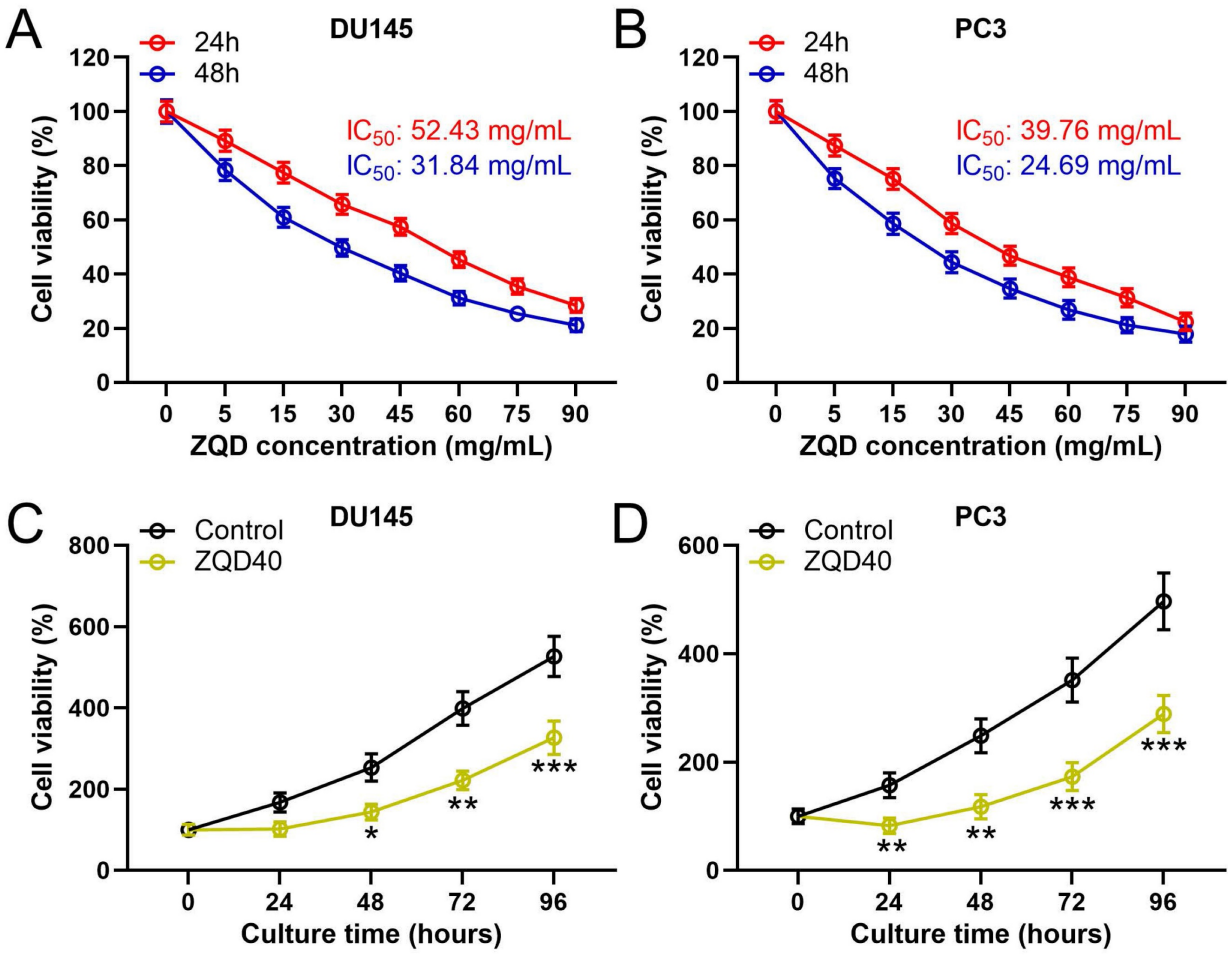
ZQD inhibited cell proliferation of PC3 and DU145 cells. DU145 and PC3 cells were treated with the specified concentrations of ZQD for 24 and 48 h, and then subjected to MTT assay and the cell viability was compared (A and B). IC50 was calculated from the curves. DU145 and PC3 cells were treated with 40 mg/mL ZQD for different times, MTT was used to measure the cell viability in different time (C and D). Results are expressed as means ± SD. n = 6. *p < 0.05, **p < 0.01, ***p < 0.001 compared to control. Unpaired t test with Welch's correction.

To examine the cytotoxicity of ZQD, PC3 and DU145 cells were treated with different concentrations of ZQD for 24 and 48 h. We found that ZQD inhibited PC3 and DU145 cell viability in a dose-dependent manner (Figures 1A, 1B). The IC50 values of ZQD for DU145 cells were 52.43 and 31.84 mg/mL when treated for 24 and 48 h, respectively. Similarly, the IC50 values of ZQD for PC3 cells were 39.76 and 24.69 mg/mL when treated for 24 and 48 h, respectively. Moreover, treatment with ZQD at a concentration of 40 mg/mL remarkedly inhibited cell viability in a time-dependent-manner (Figure 1C, 1D). Overall, our data suggested that ZQD effectively inhibited PC3 and DU145 prostate cancer cells viability.

### ZQD inhibited IL-6 induced cell proliferation of PC3 and DU145 cells

**Figure 2:**
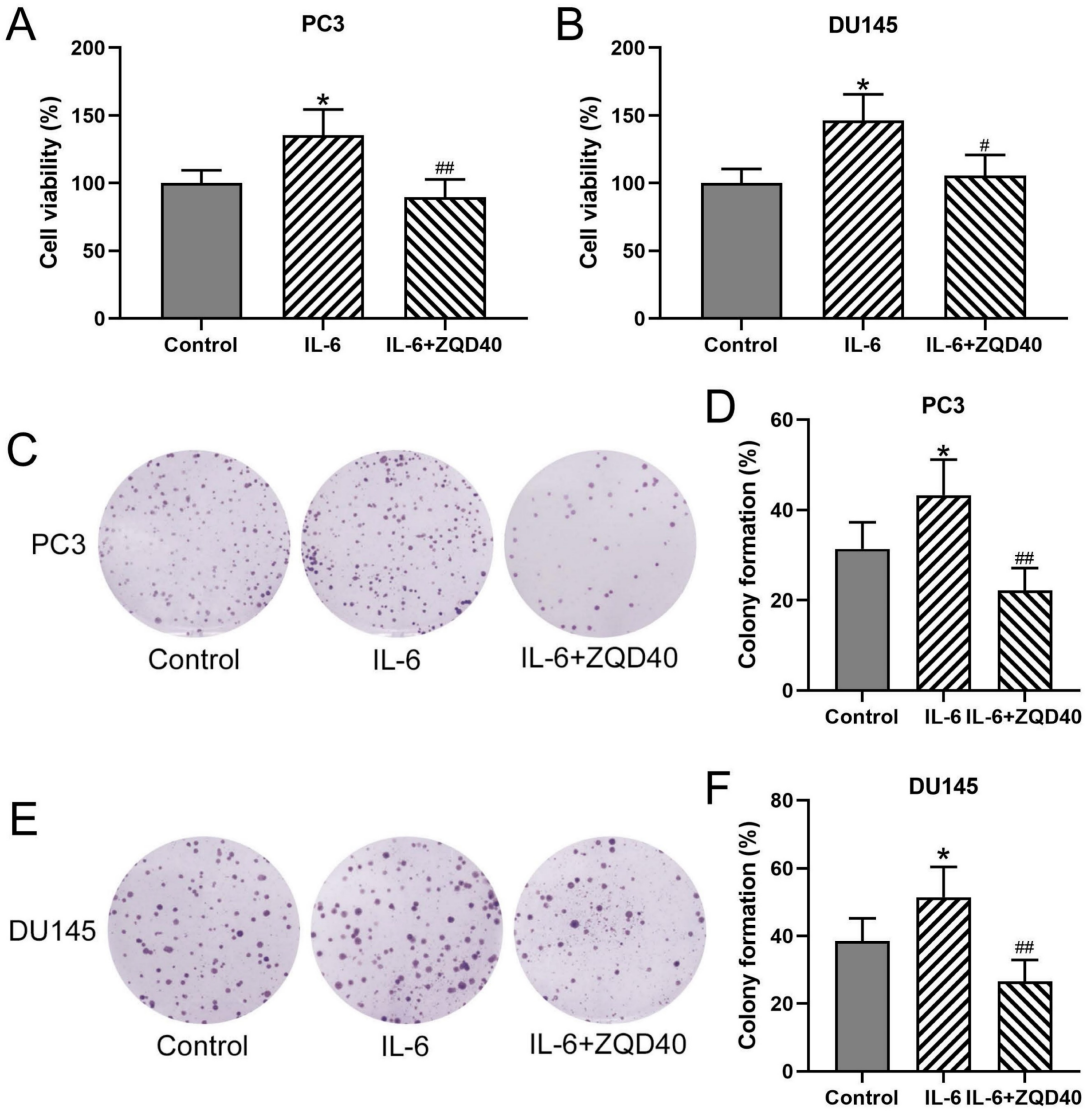
ZQD inhibited IL-6 induced cell proliferation of PC3 and DU145 cells. PC3 and DU145 cells were subjected to IL-6 (40 ng/mL) stimulation and cultured with 40 mg/mL ZQD for 24 h. MTT was used to measure the cell viability (A and B). The colony formation assay was conducted 10 days after the stimulation (C and E). The relative colony formation was compared (D and F). Results are expressed as means ± SD. n = 6. *p < 0.05 compared to control. #p < 0.05, ##p < 0.01 compared to IL-6 group. Brown-Forsythe ANOVA test followed Dunn's multiple comparisons test.

To determine whether ZQD can inhibit cell proliferation, PC3 and DU145 cell proliferation were stimulated using IL-6. As shown in Figure 2A and 2B, cell viability of both PC3 and DU145 were significantly increased after IL-6 stimulation for 24 hours compared to control (both P < 0.05), which was remarkedly reduced by ZQD treatment at 40 mg/mL (P < 0.05, P < 0.01 for PC3 and DU145 cells, respectively). Next, to confirm the inhibitory effect of ZQD on cancer cells proliferation, the colony formation assay was performed in PC3 and DU145 cells. A significant increase was found in colony formation in both PC3 and DU145 cells with IL-6 treatment for 24 hours (Figure 2C-2F, both P < 0.05). Notably, treatment with ZQD at 40 mg/mL significantly reduced colony formation when compared to IL-6 treated group (Figure 2C-2F, both P < 0.01). Overall, these data suggested that ZQD treatment at 40 mg/mL inhibited cell proliferation effectively.

### ZQD inhibited IL-6 induced IL6/STAT3 pathway activation of PC3 cells

**Figure 3:**
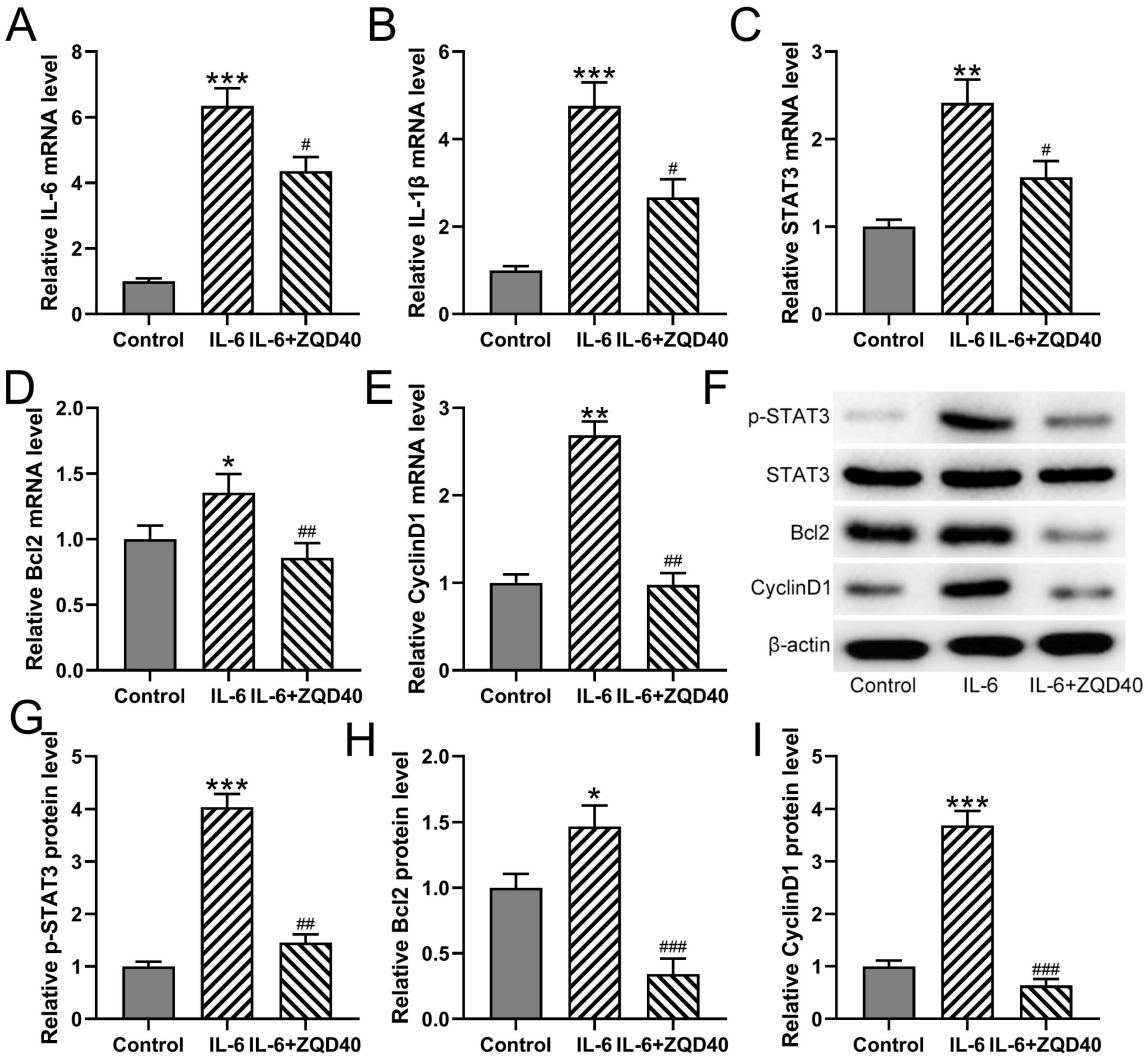
ZQD inhibited IL-6 induced IL6/STAT3 pathway activation of PC3 cells. PC3 cells were subjected to IL-6 (40 ng/mL) stimulation and cultured with 40 mg/mL ZQD for 24 h. qRT-PCR was used to measure the mRNA levels of IL-6, IL-1β, STAT3. Bcl2 and CyclinD1 (A-E). The protein levels of p-STAT3, STAT3, Bcl2 and CyclinD1 were analyzed by Western blot (F). The expressions were normalized to control (G-I). Results are expressed as means ± SD. n = 3. *p < 0.05, **p < 0.01, ***p < 0.001 compared to control. #p < 0.05, ##p < 0.01, ###p < 0.001 compared to IL-6 group. Brown-Forsythe ANOVA test followed Dunn's multiple comparisons test.

Furthermore, it was found that mRNA levels of IL-6, IL-1β, STAT3, BCL2, and CyclinD1 were significantly upregulated when stimulated with IL-6 (Figure 3A-3E). In contrast, ZQD treatment at 40 mg/mL effectively suppressed the mRNA expression of all of them. In parallel, the protein levels of p-STAT3, BCL2, and CyclinD1 were increased in IL-6 treated group when compared to the control group. All of these proteins were significantly reduced by ZQD treatment at 40 mg/mL (Figure 3F-3I). Overall, these findings indicated that ZQD inhibited the activation of IL6/STAT3 signaling in PC3 cells.

### ZQD inhibited human prostate cancer cell growth in vivo

**Figure 4:**
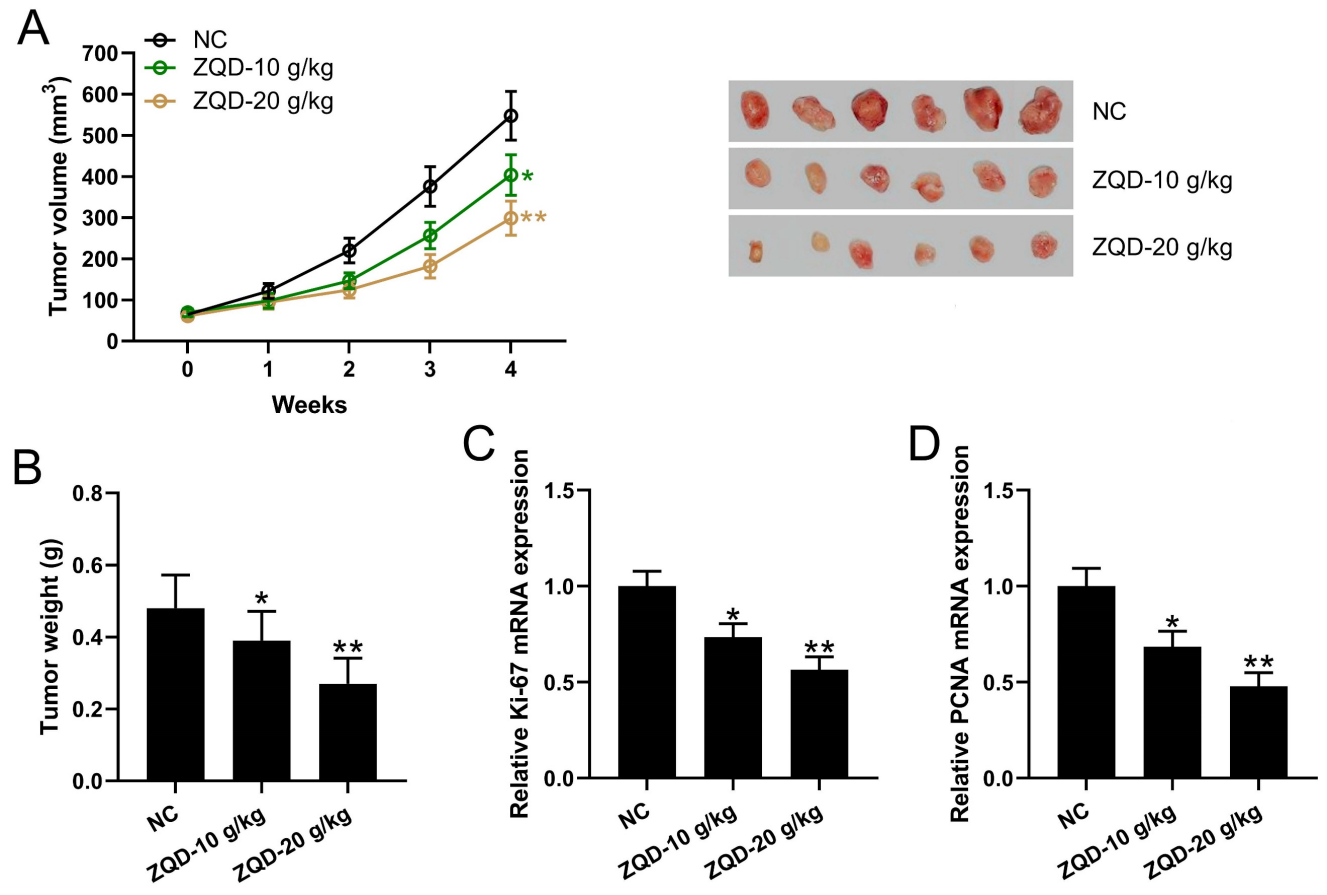
ZQD inhibited human prostate cancer cell growth in vivo. PC3 xenograft tumor model was set up in Balb/c nude mice. The mice were treated with ZQD for 4 weeks. The tumor growth curve (A) and tumor weight at day 28 (C) were shown. n = 6. qRT-PCR was used to measure the mRNA levels of Ki67 (C) and PCNA (D) in the tumor homogenate from each group. n = 3. Results are expressed as means ± SD. *p < 0.05, **p < 0.01 compared to NC. Brown-Forsythe ANOVA test followed Dunn's multiple comparisons test.

We calculated the tumor volumes at different time points and weighed the tumor tissues after 4 weeks using PC3 xenograft tumor model. We found that tumor volume and weight were significantly reduced in the ZQD-L group when compared to control group (Figure 4A-4B). The tumor volume and weight were further reduced in the ZQD-H group (Figure 4A-B). mRNA levels of Ki67 and PCNA in the tumor tissues were reduced by ZQD treatment both at low and high doses (Figure 4C-4D). In parallel, the mRNA expression of PCNA were measured in PC3 and DU145 cell lines. PC3 and DU145 cells were subjected to IL-6 (40 ng/mL) stimulation and cultured with 40 mg/ml ZQD for 24 h. Together, these results indicated that ZQD inhibited tumor growth and proliferation effectively.

### ZQD inhibited IL6/STAT3 pathway of human prostate cancer cell in vivo

**Figure 5:**
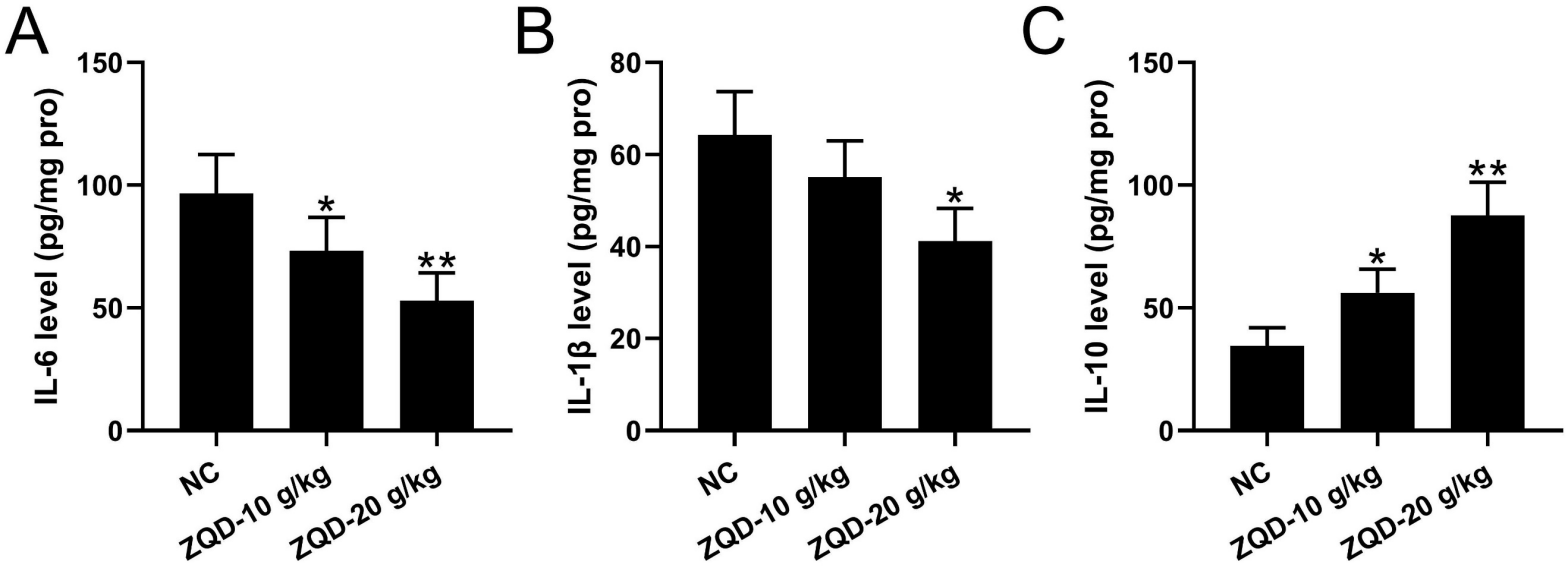
ZQD inhibited IL6/STAT3 pathway of human prostate cancer cell in vivo. PC3 xenograft tumor model was set up in Balb/c nude mice. The mice were treated with ZQD for 4 weeks. The concentrations of IL-6 (A), IL-1β (B) and IL-10 (C) in the tumor tissues were measured by ELISA. n = 6. Results are expressed as means ± SD. *p < 0.05, **p < 0.01 compared to NC. Brown-Forsythe ANOVA test followed Dunn's multiple comparisons test.

**Figure 6:**
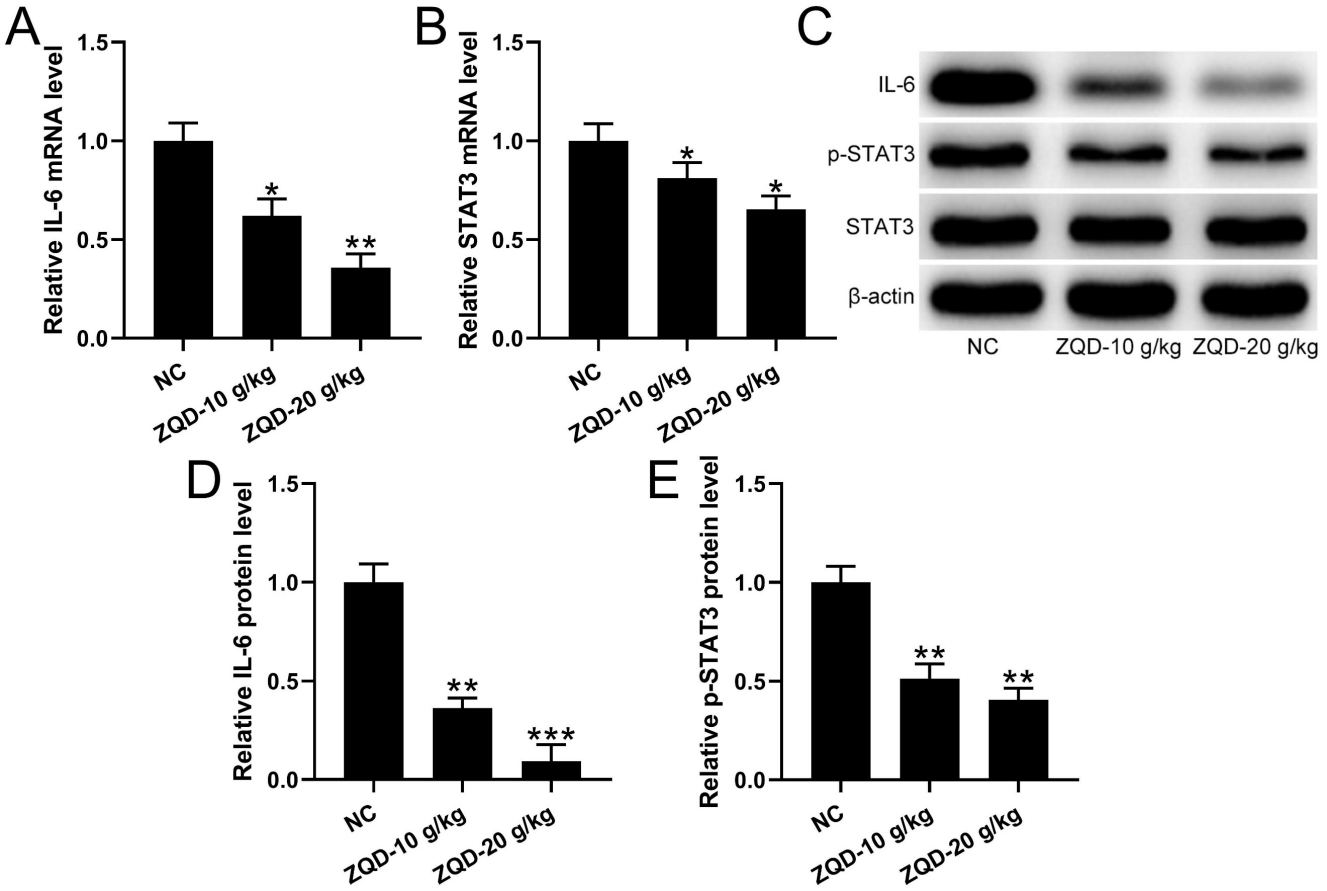
qRT-PCR was used to measure the mRNA levels of IL-6 (A) and STAT3 (B) in the tumor homogenate from each group. n = 3. The protein levels of IL-6, p-STAT3, STAT3 in the tumor homogenate were analyzed by Western blot (C). The expressions were normalized to NC (D-E). n = 3. Results are expressed as means ± SD. *p < 0.05, **p < 0.01, ***p < 0.001 compared to NC. Brown-Forsythe ANOVA test followed Dunn's multiple comparisons test.

To evaluate the effect of ZQD on IL6/STAT3 signaling pathway, we measured cytokines and STAT3 phosphorylation in PC3 xenograft tumor. We found that the levels of IL-6, IL-1β were reduced, while IL-10 was increased by ZQD treatment at high dose when compared to control group, while ZQD treatment at low dose could significantly decrease the IL-6 level, while increase the IL-10 level when compared to control group (Figure 5A-5C). Moreover, the mRNA and protein levels of IL-6 and p-STAT3 were all significantly reduced by ZQD treatment both at low and high doses when compared to the control group (Figure 6A-6E). Together, these data indicated that ZQD remarkedly inhibited the activation of IL6/STAT3 signaling pathway in vivo.

## Discussion

Prostate cancer is one of the most common malignant tumors in men, endangering their health and lowering their quality of life 3. Previously, we have, for the first time, demonstrated the efficacy of ZQD in improving survival of prostate cancer patients 16. However, the mechanism of ZQD in inhibiting prostate cancer cell proliferation and tumor growth remain unclear. In this study, prostate cancer cells PC3 and DU145 and PC3 xenograft model were used to further investigate the mechanism involved. We demonstrated that ZQD regulated the IL6/STAT3 signaling pathway on the tumor microenvironment and thus inhibits the proliferation of prostate cancer cells.

IL-6 mediates an increase in tumor cell malignancy, which may be related to its activation of the STAT3 signaling pathway. It is an oncogene that promotes cancer cell proliferation, migration ability, reduces apoptosis, and decreases adhesion 19,20, thereby increasing the malignancy and aggressiveness of tumor cells. In a basic study on prostate cancer, IL-6 was found to be highly expressed in androgen-resistant prostate cancer cell lines (DU145, PC3) and hardly expressed in androgen-dependent prostate cancer cells (LNCaP). It was found to be highly expressed in the tumor tissues of prostate cancer patients who underwent radical prostatectomy and in the serum of advanced androgen-resistant prostate cancer patients 21-23. Also, IL-6 was found to be highly expressed in the tumor tissues of prostate cancer patients undergoing radical prostatectomy and in the sera of patients with advanced androgen-resistant prostate cancer 10,20,11. IL-6 is secreted by tumor cells to regulate tumorigenesis and progression in two ways: it binds to IL-6 receptors (IL-6R) on its own target cells through the autocrine pathway, and it acts on IL-6R on the surface of other cells through the paracrine pathway 24. Kobayashi et al. showed that IL-10 levels and tumor differentiation were associated with lymph node, local and distant metastasis in prostate cancer, which may be related to the suppression of immune response by IL-10 through the inhibition of IL-12 and γ-interferon (IFN-γ) 25.

It was found that IL-6 could inhibit the oncogenic and cell cycle protein A-mediated phosphatidylinositol 3 kinase signaling pathway on prostate cancer cells by inhibiting the anti-apoptotic effect of the gene Mcl-1 after fine expression 26,27. Dong et al. found that IL-6 downregulated the expression of programmed cell death factor 4 gene in prostate cancer cells, thereby inhibiting apoptosis. IL-6 plays an important role in promoting proliferation and anti-apoptosis in prostate cancer cells by upregulating or inhibiting the expression of various signaling pathways in prostate cancer cells 28.

STAT3 is a signal transducer and activator of transcription that transmits signals from outside the cell to the nucleus, including cell growth, proliferation, differentiation, apoptosis, and central nervous system development. Recent studies have shown that STAT3 is involved in the development of various tumors and is considered to be an oncogene 29,30. The phosphorylation level of STAT3 was significantly increased in tumor tissue samples from prostate cancer patients with high Gleason score 31. It was further demonstrated that heat shock protein 27 increased the metastatic and invasive ability of prostate cancer cells by upregulating the phosphorylation level of STAT3 in prostate cancer cells, which led to epithelial mesenchymal transition 32. In a study by Liu et al., it was found that sustained activation of STAT3 in prostate cancer cells led to the emergence of androgen-resistant prostate cancer, while downregulation of STAT3 activation levels resulted in prostate cancer cells being sensitive to chemotherapeutic agents again 15. Recent studies have shown that STAT3 signaling can promote angiogenesis by regulating VEGF-A and MMP2 expression, while inhibition of STAT3 activation can inhibit tumor angiogenesis and growth 33.

There are a few limitations in the current study. Firstly, the inhibitory effects of ZQD on prostate cancer cells invasion and metastasis has not been confirmed in this study. Secondly, sample size in the current study is not strong enough. Robust results can be obtained by increasing sample size to increase the power of the study. Lastly, JAK/STAT3 signaling pathway can be activated by the production of IL-6. In order to obtain a more comprehensive effects of ZQD on IL-6/STAT3 pathway, the activation of upstream regulator JAK can be included.

## Conclusion

In this study, we investigated the mechanism of ZQD in inhibiting prostate tumor proliferation using both in vitro and in vivo models. We demonstrated the efficacy of ZQD treatment in reducing prostate cancer cells proliferation and tumor growth, evidenced by inhibiting the activation of IL-6/STAT3 signaling pathway *in vitro* (PC3 and DU145 cells) and xenograft tumor model *in vivo*. These data provide the evidence to support that ZQD can act as a therapeutical pharmacological treatment for advanced prostate cancer.
